# Advances in hPSC expansion towards therapeutic entities: A review

**DOI:** 10.1111/cpr.13247

**Published:** 2022-05-31

**Authors:** Shelly E. Tannenbaum, Benjamin E. Reubinoff

**Affiliations:** ^1^ The Hadassah Human Embryonic Stem Cell Research Center, The Goldyne Savad Institute of Gene Therapy Hadassah Hebrew University Medical Center Jerusalem Israel; ^2^ Department of Obstetrics and Gynecology Hadassah Hebrew University Medical Center Jerusalem Israel

## Abstract

For use in regenerative medicine, large‐scale manufacturing of human pluripotent stem cells (hPSCs) under current good manufacturing practice (cGMPs) is required. Much progress has been made since culturing under static two‐dimensional (2D) conditions on feeders, including feeder‐free cultures, conditioned and xeno‐free media, and three‐dimensional (3D) dynamic suspension expansion. With the advent of horizontal‐blade and vertical‐wheel bioreactors, scale‐out for large‐scale production of differentiated hPSCs became possible; control of aggregate size, shear stress, fluid hydrodynamics, batch‐feeding strategies, and other process parameters became a reality. Moving from substantially manipulated processes (i.e., 2D) to more automated ones allows easer compliance to current good manufacturing practices (cGMPs), and thus easier regulatory approval. Here, we review the current advances in the field of hPSC culturing, advantages, and challenges in bioreactor use, and regulatory areas of concern with respect to these advances. Manufacturing trends to reduce risk and streamline large‐scale manufacturing will bring about easier, faster regulatory approval for clinical applications.

## Graphical abstract

Dynamic suspension culture systems in the form of bioreactors, unlike static ones, can overcome unfavourable environmental culture conditions, assisting hPSCs to remain pluripotent and undifferentiated, or promoting their differentiation and expansion to desired cell types. They reduce medium consumption and workload, have high scalability, and allow easy online sampling for quality control analysis or other needed testing. Depending on the type of bioreactor chosen, their use permit robust expansion of large‐scale hPSCs with high‐quality, relatively homogeneous cultures, and controlled production to meet manufacturing needs for clinical trials. Closed, single‐use, well‐monitored, minimally manipulated systems will easier meet regulatory standards in bringing hPSC therapies to the clinics.

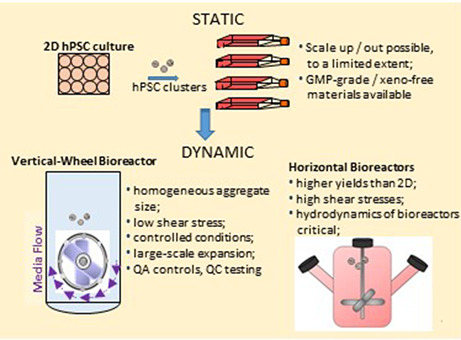

## 1 INTRODUCTION

Human pluripotent stem cells (hPSCs), which include human embryonic stem cells (hESCs) and human induced pluripotent stem cells (hiPSCs), have two essential characteristics in common: they can self‐renew indefinitely in culture, and can become almost any cell type in the body.^1^, ^2^ The power of hPSCs is that they are most versatile: they may be utilized for disease modelling and in basic scientific research, drug screening and drug development, in toxicity studies, and may serve as unlimited renewable sources of cells for transplantation therapy.

The potential for using hPSCs in cellular therapy and regenerative medicine is well known: hPSC‐derived differentiated product may be a source of therapeutic cells and restore function by direct cell replacement, once the cells are delivered directly to the sites of pathology and engraftment occurs. Both hESCs and hiPSCs can be differentiated to give rise to distinct cell types (such as neurons, beta cells, or retinal pigmented epithelial cells, for example) for use in clinical applications. Alternatively, precursor cells, differentiated tissues, or organs may be transplanted with the goal of restoring function in human patients. hPSCS can be utilized to study the genetic mechanisms of diseases. Human iPSCs derived from disease‐affected cells can be exploited to recapitulate mechanisms of disease processes that may lead to the development of detection tools and treatments for human diseases. hiPSC‐derived disease models can be also useful in screening therapeutic compounds that may then treat specific‐patient populations affected by the disease. hPSCs may be cultured either as two‐dimensional (2D) monolayers, as three‐dimensional organoids or as organs‐on‐a chip, which then can be used as tools in learning about disease phenotypes, and on which to test therapeutic agents. hPSC‐derived differentiated cells can also be used for in vitro toxicology studies and subsequent drug screening. For example, they may be expanded to generate glucose‐responsive insulin secreting β‐like cells for high‐throughput toxicity screening to identify potential β‐cell toxins. Organoid and hiPSC‐derived models may be expanded in vitro, and can be used for drug safety and screening studies as well as exploring potential toxicological effects of drugs in lieu of animal testing.^3^

Traditionally, drug discovery and toxicology studies have relied heavily on animal testing of a limited number of compounds. Such processes raise ethical concerns related to animal experimentation, are costly, and time‐consuming. Reproducible production of hPSC‐derived differentiated cells of specific types now may allow high‐throughput screening (HTS) for therapeutic or toxic effects of hundreds or thousands of candidate molecules in short periods of time, at relatively low cost. However, to do so, the barriers of mass‐production must be overcome.

An efficient means of translating hPSCs into scalable cell numbers require an alternative to manual cell‐culturing methods. Traditionally, these manual methods were low‐scale, labor intensive, and highly variable. Over time, improvements in culture methods, such as the use of enzymatic passaging, feeder‐free and defined culture systems, and the utilization of bioreactors improved scalability, lowered costs, and improved efficiency.^4^ Moreover, robotic platforms^5^, ^6^ and automated approaches might further promote rapid, standardized homogeneous manufacturing of billions of cells for eventual use in drug screening, toxicology, disease modelling, and cell therapy. These advances, combined with currently used robotic automated systems for HTS and toxicology, may allow fully‐automated, large‐scale, controlled, and reproducible processes.^7^

The understanding of the human body and embryogenesis benefited from the use of hPSCs in the study of embryonic development.^8^ Numbers of cell‐therapy products entering clinical trials every year grows exponentially, requiring ever‐growing batch sizes. Reports show that at the end of 2019, there were at least 54 ongoing clinical trials based on hPSCs, aimed at treating 22 different diseases. These cell‐therapy treatments include 32 from hESC‐derived products and 19 from hiPSC‐derived ones.^9^ The industry potential supply simply no longer can keep up with clinical need, at this rate. Functional hPSC progenies are mainly generated by protocols of research‐grade scale and quality. While attempts have been made to utilize these cells in early clinical applications, large industrial scale numbers under clinical‐grade conditions will be necessary to meet regulatory and industry needs. Rough estimations suggest that for replacing disease‐induced loss of cells, such as hepatocytes, pancreatic‐cells, or cardiomyocytes, approximately 1 × 10^9^–10^12^ functional cells per patient,^10^ depending on the therapeutic target, will be required. Higher numbers, in the case of red blood cells for in vitro transfusion have been mentioned (2.5 × 10^12^ cells^11^). Additionally, 10^10^ cells may be required for typical pharmacological or toxicological screens.^12^ Moreover, large cell numbers are also necessary ahead of treating patients: for proof‐of‐concept studies in small (rodents) and in large animals (pigs or non‐human primates). Therefore, to use hPSCs in regenerative medicine will necessitate large‐scale production processes by standardized, GMP‐grade, and economically viable procedures and technologies.

## 2 CURRENT PLATFORMS & THEIR CHALLENGES

To enable their widespread use in regenerative medicine, hPSCs should be cultured and expanded on a scale larger than ever before. While hPSC culturing in two‐dimensional (2D) systems traditionally were carried out in culture dishes on mouse embryonic fibroblast feeders,^2^, ^13^ use of mitotically‐inactivated feeder layers and static 2D culture systems are neither stable, scalable, nor cost‐effective. Feeder‐free and xeno‐free options for culturing hPSCs were therefore developed. These alternatives (Table 1) increased cell safety by avoiding contaminating cultures with unknown proteins, prions and zoonotic viruses, and improved growth efficacy and scalability limitations using new biological technologies for coating culture plates to promote cell growth, maintenance, or differentiation.^14^ While 2D scale‐up (increase in cell numbers or yield by using larger culturing vessels) and scale‐out (increase in cell numbers or yield by manufacturing in parallel vessels) became reasonable with the introduction of CellSTACKs®, the use of these cell factories still have limitations that make them challenging to use for commercial or even large clinical‐trial manufacturing batch runs. They require considerable incubator space, visualization of the cells is limited, and passaging may be challenging.

**TABLE 1:** Advantages and disadvantages of hPSC culture systems

Culture system			Advantages	Disadvantages
2D	On feeders		Monolayer cultures accessible to visualization Can scale‐up Cells evenly exposed to medium & components	Visualization of cells is limited (CellStacks®) Labor intensive, not easily scalable Not cost‐effective Batch‐to‐batch variation
	Feeder‐free, Xeno‐free		Monolayer cultures accessible to visualization Reagents used may increase safety Increased growth efficacy and scalability	Visualization of cells is limited (CellStacks®) Scale‐out is limited Requires much incubator space
3D	Static	Adherent conditions	Allow better scale‐up & scale‐out Simple medium exchange Use of microcarriers & hydrogels lead to uniform cluster formation and increase in yields	Space limitations (depending on platform) Variability in hPSC viability, expansion yields, homogeneity, and differentiation capacities
	Dynamic (Bioreactors)	General	Larger models have online monitoring and sampling for QC analysis, control of process parameters Shortened culture time, high scalability Reduced medium consumption; control of hPSC aggregates Prevention of formation of gradients by supporting more homogeneous distribution of culture components More consistent dissolution of nutrients and gases Facilitate development of large‐scale hPSC processes with high cell densities	Aggregates can settle at the bottom of the vessel in horizontal‐blade bioreactors in dead zones of high shear stress High shear stresses can split or damage cells or aggregates Shear stresses can affect yields or quality of cells
	Horizontal bioreactors		Homogeneous mixing, low shear stresses, good distribution of nutrients and gases Most commonly used type of bioreactor due to availability, ease of setup, enhanced yields with microcarriers	Limited scalability due to size restraints Cell growth rate compromised due to fluid shear stress turbulence Pitched impeller blades cause spiral mixing, leading to uneven particle suspension and heterogeneous aggregate size
	Spinner flask			
	Slow turning lateral vessel (STLV)		Low fluid shear stress Increased oxygenation levels by diffusion Useful in EB formation; homogeneous aggregate development	Scaling options limited due to size and space
3D	Stirred tank bioreactors		Efficient mixing Impeller rotation keeps cells in suspension	Scale‐up is limited High shear forces are caused by impellers lead to smaller hPSC aggregates, which affect pluripotency and viability
	Rotating wall bioreactors (RWB)		Low shear stresses on cells due to horizontal, continuous rotation of vessel	Cell harvesting and process monitoring restricted to pauses in production Scale‐out limited
	Wave bioreactors		Can be scaled‐up Larger models can be automated	Scale‐out limited Need large space to operate
	Vertical bioreactors		Mixing in an infinity‐like motion, which is favourable for hPSC growth Less shear stress for hPSC expansion More uniform, limited‐sized hPSC aggregate formation Scalable Larger bioreactors have standard monitoring probes & process controllers	Smaller volume bioreactors lack monitoring probes & process controllers
	Vertical‐wheel			

Suspension culturing and other three‐dimensional (3D) culture systems better utilize the space available for hPSC proliferation and differentiation. They allow scale‐up and scale‐out to meet the challenges of today's clinical needs. Depending on the type and size of bioreactor (Table 1) and media chosen, bioreactors have the benefits of online monitoring and control of process parameters (such as pH, dissolved oxygen, pCO2), as well as generally shortening cultivation time.^15^

### 2.1 2D culture systems and process scale‐up

Conventional 2D culture systems normally consist of plating hPSCs on culture dishes or flasks (on either mitotically inactivated human or mouse embryonic fibroblasts). Extracellular matrix proteins such as fibronectin, Matrigel®×, or laminin may be used to coat the plates if feeder‐free growth is desired. Fibroblast‐conditioned or chemically‐defined media supplemented with growth factors and other proteins may be used to maintain pluripotency. Advantages for some of these well‐established 2D systems in flasks and plates may include (Table 1) their ease‐of‐use, effortlessness visualization, and relative low cost. Disadvantages include that they may be labor‐intensive, manual passaging may cause batch‐to‐batch variations, and have limited scale‐out capabilities.^4^ 2D culture systems may be scaled‐up, but not easily scaled‐out. That is, by increasing the size of the flasks, the number of wells seeded, or with the implementation of cell factories (CellStacks®) larger batches may be produced—however, considerable challenges remain. These platforms require significant amounts of space and human interaction, which would make them impractical for large‐scale expansion. This platform was used to derive the first known GMP‐grade, xeno‐free banks of clinical‐grade hESCs.^16^ While several new clinical‐grade hESC lines were derived, the batch sizes were relatively limited due to space and manufacturing limitations. Whereas automation of 2D cultures is possible with the newer technologies, such as computerized cell factory manipulators (ThermoFisher Scientific), these systems would still require substantial space to generate production scales of hPSCs for therapeutic applications.

### 2.2 3D–Suspension (static)

3D hPSC culture methods allow significant scalability to meet therapeutic demands. 3D methods include static suspension culture, with or without the use of encapsulation‐based hydrogels or microcarriers. Scale‐out takes existing infrastructure and replicates it to work in parallel to increase cell yields. Advantages to using these systems include easy scale‐up of culture volume and simple medium changes.^17^ However, there are differences in hPSC viability, expansion yield, homogeneity, differentiation capacity, and its applicability to industry.^18^ Whereas static processes, including both single‐cell and cluster culturing of hPSCs in suspension may be simple and robust, they may not be suitable for large‐scale regenerative therapy applications because of scale‐out limitations, since they require much incubator space. Steiner et al. showed,^19^ for the first time, the derivation and long‐term propagation of hESCs under suspension culture conditions. The hESCs remained pluripotent, maintained normal karyotypes, and could be directed to differentiate into somatic cells. Nevertheless, large‐scale expansion as well as uniformity of cell clusters were not demonstrated in this publication. Single‐cell inoculation in suspension cultures could effectively control the size of hPSC aggregates, whereas cluster inoculation is challenging due to the difficulty in controlling the size of hPSC clusters; this leads to increased apoptosis or spontaneous differentiation.^20^

Scale‐up under 3D culture conditions can benefit from utilizing microcarriers and microencapsulating hydrogels. Microcarriers increase surface area, hPSC attachment and proliferation, gas diffusion, reduce the need for culture medium and growth factors, and are discussed extensively elsewhere.^20^ Fully defined, xeno‐free options are available and were shown to modestly reduce doubling time and impact seeding efficiency.^12^ However, hESCs must be harvested from the microcarriers using chemical or mechanic means, reducing cellular viability during passaging and harvest. Microencapsulation into polymer matrices involves mixing hPSCs homogenously with a hydrogel, either as single cells or as cell aggregates. A gelation process encapsulates the cells into the hydrogel. This step condenses the cells into a semi‐solid phase, which better allows the hPSCs to withstand adverse agitation shear stresses used in 3D bioreactor systems. Like with the use of microcarriers, advantages include increase in hPSC attachment and surface area while decreasing agglomeration and shear stresses. Disadvantages consist of challenges in retrieval of the cells at the conclusion of the process, and potential diffusion limitations within the polymer gel that could affect differentiation and survival.^12^ Thermosensitive polymer hydrogels have been suggested as a solution for cell‐retrieval issues. These hydrogels utilize temperature changes to prompt their gelation and return to liquid, releasing the cells without the need to use additional factors.^21^

### 2.3 3D–Bioreactors (dynamic)

Dynamic suspension culture systems in the form of bioreactors, unlike static ones, can help overcome unfavourable environmental conditions, which assist hPSCs to remain pluripotent and undifferentiated, or promote their differentiation and expansion to desired cell types. They reduce medium consumption, have high scalability, and allow easy online sampling for quality control analysis or other needed testing. Other advantages of bioreactors (Table 1) include control of hPSC aggregation, and the prevention of the formation of gradients by supporting a more homogeneous distribution of culture components.^10^ The use of bioreactors allows a more consistent dissemination of nutrients and gases into the culture medium and, therefore, into cells or clusters, and facilitate the development of large‐scale hPSC processes at high cell density.

Finally, as manufacturing of hPSCs and cell banks becomes more automated, bioreactor systems that are equipped for process monitoring and control of the culture environment and concentrations of nutrients, metabolites, and gases can critically influence expansion, proliferation, and growth of hPSCs and their differentiated progeny. Nevertheless, the high‐shear stresses caused by bioreactors can split or damage cells or aggregates, affecting both batch yield and quality.

The types, sizes, scalability of the bioreactors chosen, direction of rotation of impeller blades and flow of medium are all crucial to generating sufficient numbers of cells for use in transplantation therapies. Horizontal blade bioreactors and vertical‐wheel systems each have advantages and drawbacks. Batch‐feeding strategies can greatly affect the fold expansion rates, and, therefore, should be carefully examined and deployed based on the specific bioreactor chosen. Fed‐batch cultures are ones where the culture medium, nutrients, and growth factors are supplied at the beginning of the process. Nutrients and growth factors are furnished throughout the run, and the products are maintained in the bioreactor until the end of the run. Perfusion cultures (also known as continuous medium replacement) are ones where culture medium, nutrients and growth factors are continuously supplied while waste medium is removed. By focusing and choosing the correct feeding system for each specific bioreactor, hPSC pluripotency, differentiation potential and process‐dependent energy metabolism may be affected.

#### 2.3.1 Horizontal bioreactors: Types, advantages & disadvantages

**FIGURE 1:**
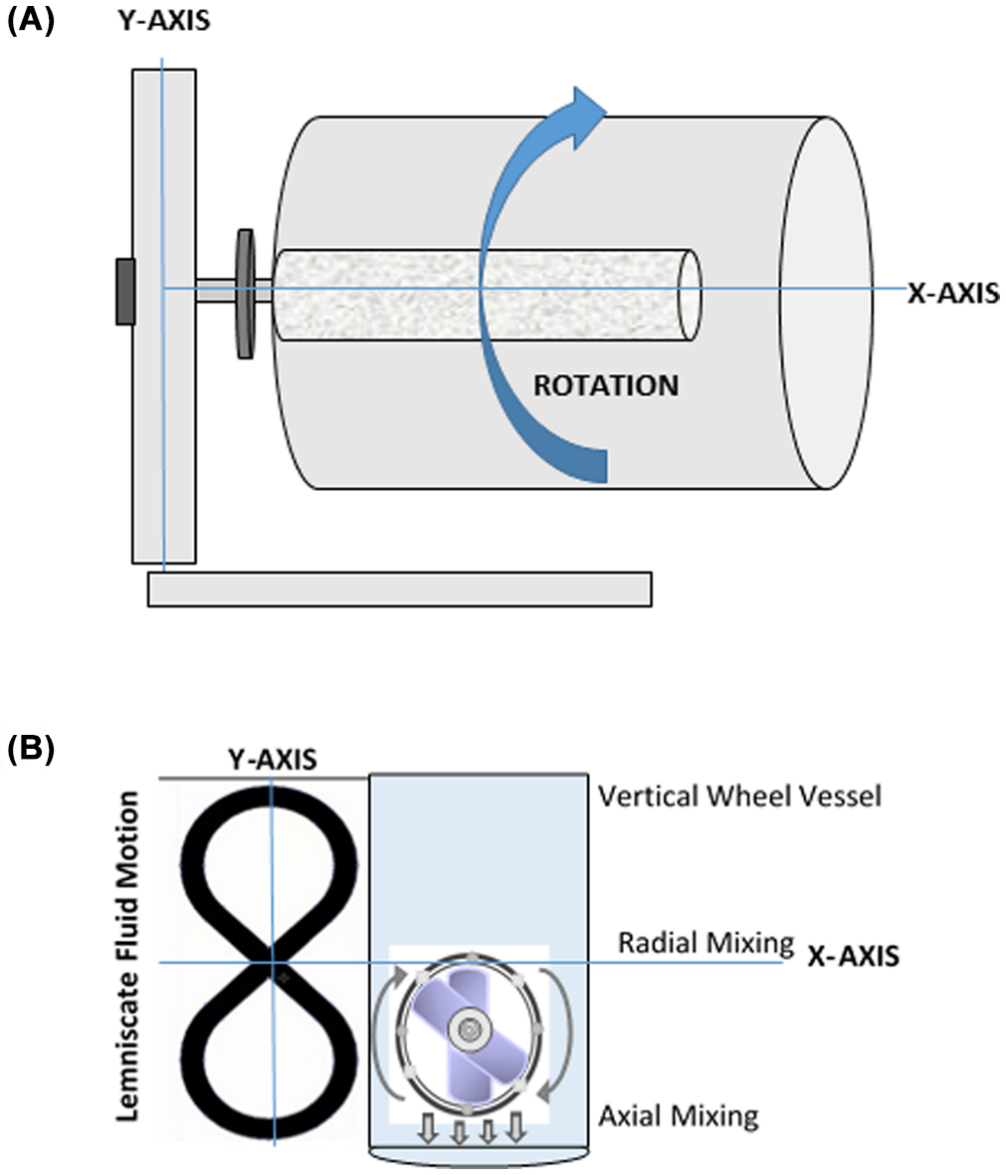
Horizontal flow and vertical‐wheel bioreactors used in hPSC expansion. (A) Rotating wall bioreactors (RWBs) continuously rotate along their longitudinal, horizontal axis, creating low shear stress and homogeneous mixing. (B) Vertical‐wheel bioreactors have simultaneous radial and axial mixing, creating uniform hESC aggregates and gentle mixing

The use of bioreactors where the hPSCs are suspended in 3D dynamic culture systems is a significant advance towards scale‐up to meet clinical and manufacturing needs. Dynamic bioreactor system classifications can mainly be divided into mechanical and hydraulically driven based on the power input. The most common types are mechanically driven: they include stirred tank bioreactors (and spinner flasks), rotating‐wall bioreactors (for differentiation), or wave‐mixed bioreactors (for tissue engineering), among others.^22^ Rotating‐wall bioreactors (RWB, Figure 1A) are suspension culture systems where cylindrical, horizontally oriented culture vessels continuously rotate longitudinally, creating homogeneous mixing and low shear stresses (Table 1). Wave bioreactors (with or without microcarriers) are another culture system most commonly used in hPSC expansion. These bioreactors use rocking motions to agitate the cells, providing good distribution of nutrients and gases, and low shear stress. Both RWB and wave bioreactors have limited scalability due to size restraints. For both systems, optimization scale‐out of the process would increase hPSC yield.

Orientation and agitation of bioreactor impellers determine the direction and movement of the media flow within bioreactors. Impeller blade placement and positioning within the system can affect the homogeneous mixing of cells, nutrients, and gases within bioreactors. In traditional horizontal‐blade bioreactors, the mixing of the fluid occurs in radial (along the radius or x‐axis) or axial (up and down) directions along the shaft of the impeller blade.^23^

Spinner flasks or stirred‐tank bioreactor systems are widely used today in undifferentiated hPSC expansion. Their versatility (Table 1) is that the culturing system can be used for both single‐cell growth or expansion as clusters, using microcarriers or without. In suspension cultures, microcarrier use also promotes uniform cluster formation. One challenge in the spinner flask culturing system is that when aggregates form, they are not of homogenous size—leading to apoptosis and spontaneous differentiation. Watanabe et al. first showed that single‐cell inoculation may be enhanced by the addition of Rho kinase inhibitor (RI) Y‐27632, which improved clonal survival of hPSCs, and led to uniform aggregate formation.^24^ Another challenge in these systems is the cellular growth rate, which could be compromised due to areas of fluid shear‐stress turbulence caused by impeller blade movements and heterogeneous mass transport.^20^ Design of the impeller blades can impact the shear stresses put on the cells, as well as the mixing capacity of the culture contents. For example, the shape of the impeller blade in spinner flasks is generally “pitched,” i.e., they are flat and may be set at ~45° angles for shear‐sensitive mixing (for highly viscous substances), producing simultaneous axial and radial mixing.^23^ This spiral mixing leads to uneven particle suspension and heterogeneous aggregate size. Alternatively, slowly turning lateral vessel (STLV) bioreactors, a type of rotating wall vessel, have been shown to have very low fluid shear stresses and high oxygenation levels by diffusion. Researchers have demonstrated that they are useful in EB formation and homogeneous aggregate development.^25^, ^26^ Stirred tank bioreactors generally use agitation by stirring with impellers to obtain uniform mixing. The high shear forces instigated by these movements can cause smaller hPSC aggregates, affecting their pluripotency and viability.^27^ Rotating‐wall bioreactors provide large surface‐to‐volume ratios, while conveying low shear stresses to the cells due to the horizontal, continuous rotation of the vessel.^23^, ^28^ Due to the nature of this bioreactor's design, cell harvesting and process monitoring are limited during culturing. A key goal in suspension growth with bioreactors is to obtain homogeneous aggregate growth, but aggregates of limited size. Kropp et al. showed that aggregates with diameters larger than ~300 μm are known to result in cell necrosis due to limited nutrient and gas diffusion to tissue/aggregate centres.^10^ Dissociation of aggregates into single cells during passaging can support selective culture conditions for hPSCs that are more homogeneous and pluripotent, and less differentiated than those that had not been dissociated. Yet accounts exist that single‐cell enzymatic dissociation of hPSCs results in loss of cell viability.^29^ For this reason, when using bioreactors, most seeding is done as clusters, and grown as aggregates. Even cultured in this manner, the clusters may grow heterogeneously, spontaneously differentiate, and lead to cell apoptosis.^20^ There is no doubt that the effects of shear stress and the hydrodynamics of the flow within the bioreactors' culture system on hPSCs majorly affect the quality of the cells. Agitation of the impeller‐blade affects both mixing capabilities and the energy dissipation rates^29^—that is, the amount of energy lost due to viscous forces in fluids—within the system.

#### 2.3.2 Vertical bioreactors: Types, advantages & disadvantages

Unlike the horizontal‐blade bioreactor, the vertical‐wheel bioreactor (PBS Biotech, Ltd) is unique in its design, with a novel hydrodynamic mixing pattern and lower shear stress due to special fluid dynamics and a revolving wheel motion (Figure 1B). The fluid motion moves in an infinity‐like motion (i.e., lemniscate pattern^29^) throughout the bioreactor, which is favourable for hPSC growth (Table 1). In horizontal‐blade bioreactors, the mixing patterns are less favourable; more aggregates settle at the bottom, forming large conglomerates under the mixing blades, in areas typically known as “dead zones”, with associated high shear stress. In the vertical‐wheel bioreactor, due to the radial‐lemniscate pattern of mixing, and because of the U‐shaped bottom of the bioreactor vessels and associated fluid mixing patterns, less shear stress is placed on aggregates as they are swept along in fluid through the vessel and along its bottom. Jossen et al. have shown that shear stress on hPSCs can cause differentiation^22^; therefore, depending on the stage of use, higher or lower shear stress may be desired: If minimizing differentiation is required, such as during the expansion phase, lower shear stress environments are optimal. However, during differentiation, moderate‐to‐higher levels of shear stresses would be acceptable. The ideal bioreactor would be one that would be adaptable to shear stresses depending on the needs of the user, while considering the growth kinetics and behavioural characteristics of the hPSCs.

Large aggregates can form due to an increase in cell number following feeding, and a greater production of extracellular matrix.^30^ Caruso et al. theorized that these cellular growth surges occur at the end of the exponential growth phase, and cells within these large aggregates are unable to receive sufficient nutrients and oxygen and die.^31^ Therefore, for optimum cell viability and yield, aggregates should be of limited size to be viable for harvesting after expansion.

Borys et al.^29^ showed that the unique mixing pattern in the vertical‐wheel bioreactor resulted in a more uniform, limited‐sized aggregate formation. In a side‐by‐side comparison with a horizontal‐blade bioreactor, the vertical‐wheel environment supported uniform aggregate growth with healthy morphologies, high cell fold expansion, and maintenance of cell pluripotency. They were successful in generating consistent aggregate sizes from single‐cell inoculations at all tested agitation rates. The authors theorized that the radial mixing in horizontal bioreactors led to fluid dead zones, high or low shear environments, and limited the movement of aggregates through the fluid volume, causing low cell yields.

Borys speculated^29^ that in the vertical‐wheel bioreactor, the fluid mechanics of the energy dissipation rate remains homogeneous, and lower agitation rates (i.e., 40 rpm) regulate and limit aggregate size. However, in horizontal‐blade bioreactors, the aggregates flow in a mostly radial fashion along with the fluid and become trapped in alternatingly high or low shear stress zones, forming both large and small aggregates.

The vertical‐wheel bioreactor family offers models as small as 100 ml up to those meant for commercial manufacturing reaching 500 L. The novelty of the wide range of products is scalability: that is, as the maturity of the process progresses, the bioreactor advances with one's needs. The small volume bioreactor models lack process controllers, so culture parameters such as pH, dissolved oxygen, nutrient and metabolite concentrations, and temperature cannot be monitored by the bioreactor. At higher volumes, the vertical‐wheel bioreactors are equipped with standard monitoring probes and process controllers, which allow for online monitoring and are fully controlled bioreactor systems—major steps for hPSC expansion and differentiation and for their use in clinical applications.

### 2.4 Challenges to large‐scale hPSC GMP manufacturing

To exploit the potential of hPSCs for clinical and industrial use, efficient scale‐up of GMP‐grade hPSC expansion, controlled differentiation to obtain uniform populations of specific cell types, and proficient cryopreservation techniques are necessary. Standardization of the production and cryopreservation processes reduces batch variability, which leads to more controlled, uniform, homogeneous manufacturing batches—reducing lot heterogeneity and showing process robustness. Optimizing cryopreservation techniques is critical for hPSC expansion and their ultimate development to lineage‐specific differentiated cells. hPSCs are highly sensitive to cryoinjury: During cryopreservation, apoptosis may be induced, cell‐to‐cell or cell‐to‐matrix adhesion matrices may be disrupted, or reaction oxygen species (ROS) may be produced.^32^ Cryopreservation techniques should be optimized to maintain hPSC survival, expression of phenotypic markers, and viability upon thaw, as it is essential for final distribution of products.

A key challenge in developing hPSC‐derived cell products is the cost of goods (COG). The COG represents the costs to manufacture a cell product, and includes both direct (manufacturing, materials and labor) and indirect (maintenance, taxes, insurance and equipment depreciation) expenses.^7^ Estimating COGs may be challenging if the production process lacks reproducibility and lot sizes are variable. Elimination of uncertainty, increasing reliability and robustness of the process, and decreasing the risks of contamination or bottlenecks are crucial in reducing the COG.

Cellular and gene therapies are people‐ and process‐dependent and rely on aseptic processing, since they cannot be terminally sterilized. Therefore, to decrease the risks of supplying potentially non‐sterile preparations to patients, adequate quality risk management procedures should be instituted to reduce the possibility of harm. Using closed processes whenever possible, moving to automated or robotic systems, and decreasing the risk from human interactions in the culture systems are some of the means of diminishing chances of contamination.

Key to preventing uncontrolled hPSC differentiation is a thorough understanding of the processes, differentiation potentials and stresses that culturing conditions place upon the cells. Individual hPSC lines have varying differentiation efficiencies and tendencies, and no two cell lines are alike. More effort should be placed on standardizing reproducible differentiation protocols to prevent differentiation inefficiency with chosen cell lines. The removal of undifferentiated hPSCs from differentiated product would be essential in preventing possible transplantation of malignantly transformed undifferentiated hPSCs and progenitor cells.^33^ Genomic instability of hPSCs arises from replicative stress in the culture system, causing karyotypic instability in bulk cultures. This too is an additional challenge when culturing hPSCs long‐term.

## 3 REGULATORY CONCERNS FOR LARGE‐SCALE EXPANSION OF hPSCs

Regulators have always been concerned with product quality, safety and potency. There is no doubt that as hPSC culture systems advance from 2D to 3D, from static to dynamic, from low to large scale, the complexities multiply. As the number of steps from the bench to the clinic increase, the challenges at each stage in meeting regulatory guidelines also rise. On the other hand, a rule of thumb is that “the less manipulations in the process the better”. Therefore, an hPSC product manufactured, at least in part, in a bioreactor, is easier to comply with regulations since it is less “substantially manipulated”. In the US, human cells, tissues and cellular and tissue‐based products (HCT/P) are regulated by the FDA's Centre for Biologics Evaluation and Research (CBER), and in Europe, the European Medicines Agency (EMA). The FDA is concerned about stem cell‐related therapeutic products' transplantation or infusion into patients. It believes that processes that include culturing, expanding and/or adding growth factors require regulation because these processes constitute significant manipulation.^34^ Both the FDA and EMA consider risk management activities, and extensive product characterization in the form of critical quality attributes (CQA, i.e., physical, chemical, biological or microbiological properties or characteristics that should be within an appropriate limit, range or distribution to ensure the desired product's quality or safety^35^). These qualities are based on the “severity of harm” approach to the patient resulting from the failure to meet CQA (i.e., effect on safety and efficacy if these attributes are changed). Its critical process parameters (CPP, i.e., a process parameter whose variability affects a CQA, and therefore should be monitored or controlled to ensure the process produces the desired quality),^35^ product impurity data, raw materials and stability information are all crucial to understanding the process, and are things that regulators inspect in Investigational New Drug Application (IND) submission packages.

### 3.1 GMP compliance and chemistry, manufacturing and controls (CMC)

During cell manufacturing, the product's quality is ascertained by sampling at various checkpoints. Safety, in the form of screening for microbial contamination (ICH Q4B Annex 8 Step 5),^35^ endotoxin (ICH Q4B Annex 14 Step 5)^36^ and purity by testing for undifferentiated hPSCs via evaluating specific markers of interest, or for the presence of reprogramming vectors in the case of iPSCs, may be determined at different checkpoints during the process as well as at batch release. Product potency via teratoma formation, in vitro differentiation into three germ layers, PluriTest® or other means^37^, ^38^, ^39^ is essential to establish before batch release, as are viability, karyotyping, identity and characterization by flow cytometry or immunocytochemistry.^40^, ^41^ SNP arrays or whole genome sequencing (WGS, for iPSCs) may be currently performed for information only. While every organization should determine batch release criteria specific for its cell lines and products, members of international organizations such as the Global Alliance for iPSC Therapies (GAIT), the International Stem Cell Banking Initiative (ISCBI), the Centre for iPS Cell Research and Application (CiRA, Japan), and many others meet often to discuss, clarify, and establish acceptable CQA release standard limits for clinical‐grade iPSCs and hPSCs.^40^, ^42^ Such professional organizations—made up of communities of experts in the field of hPSC research—set the minimum consensus standards and promote best practices in the field. Regulators depend on the advice of experts when determining new scientific policies. What is not new and can be expected by all regulators is that emphasis will be placed on adherence to current good manufacturing practice (cGMP) guidelines throughout the entire process, especially if the product is to be used in clinical settings. This encompasses the implementation of a thorough Quality Assurance (QA) system that will enforce GMPs, and that includes a Quality Control (QC) program. The QC program is responsible for ensuring that the product meets quality, safety, efficacy and potency requirements. For clinical applications, GMP regulations apply to all aspects of the production process—from screening and approving raw material vendors and qualification of the materials (such as EudraLex Volume 4, Good Manufacturing Practice [GMP] guideline^43^; ICH Q7 GMP for Active Pharmaceutical Ingredients^44^; ISO/TS 20399‐1:2018^45^) to manufacturing and cryopreservation, labeling, packaging, storage and distribution of the final product. The CMC section of an hPSC‐based therapeutic product in IND submissions contains detailed information about all aspects of the manufacturing process and development of the cell line, raw materials, characterization results and information regarding the reprogramming vectors, plasmids and clones (in the case of iPSC‐based products), reagents, as well as validated results of warehousing and transportation studies. Regulators will want affirmation about qualification and suitability of purpose of the raw materials chosen, genetic stability of the cell product, microbial sterility and freedom from adventitious viruses and that there is no batch‐to‐batch variation in the lots intended for transplantation. Traceability, documentation and the benefit–risk ratio of the product are the keys to approval by regulators.^46^

### 3.2 Advanced therapy medicinal products and adaptation for clinical use

In 2007, the European Commission established a new class of therapeutics originating from the biopharmaceutical industry called advanced therapy medicinal products (ATMPs; Regulation EC No 1394/2007).^47^ This consists of gene therapy medicinal products (GTMPs), somatic cell medicinal products (sCTMPs), tissue‐engineered products (TEPs) and combined products (tissues or cells associated with a device).^48^ In the US, these types of modalities are termed cell and gene therapy (CGT) products, and are overseen by the Food and Drug Administration's (FDA) Centre for Biologics Evaluation and Research (CBER). ATMPs and CGTs involve substantial manipulation and industrial‐grade production processes, and must be adapted to GMP standards for use in clinical trials.

Human PSCs may be the source material for cell and tissue‐based ATMPs for clinical trials. Cell therapies have advanced from the laboratory bench to the clinic, and constitute an important part of phase I/II clinical trials. The hPSCs are first differentiated into cell populations of interest, and then are either transplanted as suspensions,^9^ after seeding onto scaffolds,^49^ as cell sheets,^50^ or implanted after differentiation to cell progenitors in combination with devices.^51^

Mesenchymal stromal cells (MSCs) are today one of the most researched and utilized type of cells, with more than 1500 ongoing clinical studies.^52^ Preparation of batches of MSC‐derived product for use in an academic clinical setting and then upgrading these existing processes to obtain GMP certification is quite challenging. This may involve the purchase of new equipment and their subsequent qualification, process validations, new raw material choices and their qualification and management, personnel training and qualification, analytical methods validation, stability and transportation studies, clinical trial product preparation runs and more. Such upgrades can require the complete overhaul of clinical‐grade manufacturing processes in order to obtain such certification,^52^ and places a significant burden on organizations if the concept of GMP is not built into the process from the ground up. Alternatively, when translating from the preclinical to the clinical arenas, it is easier to implement GMP‐compliant procedures into an organization prior to manufacturing clinical batches: In the preparation of one organization's ATMP allogeneic implant, called Pericord, which is composed of human Wharton's jelly‐derived mesenchymal stromal cells within decellularized pericardium, their implants were produced under GMP guidelines from the first human preparation.^53^

The ATMP subclass of TEPs consists of engineered cells or tissues for human therapeutic transplantation. Their manufacture is both costly and labor intensive. As of 2021, only a few preparations of this subclass have been translated from academia to the clinic and beyond.^54^ Automating manual processes during translation to GMP manufacturing can significantly enhance production scale‐up, and cut production times while lowering costs. In one example, manually processed autologous nasal septum‐derived cartilage cells cultured on a 3D carrier matrix for treatment of focal cartilage defects could theoretically be significantly improved by processing with an automated robotic system: This would potentially eliminate human interaction and decrease the risk of contamination, implement standardization and batch homogeneity, apply process controls and real‐time monitoring, and allow multiple TEPs to be manufactured in parallel.^54^

## 4 CONCLUSION: MANUFACTURING TRENDS FOR LARGE‐SCALE EXPANSION OF hPSCs

As the demand for cell‐based treatments expands in the clinics, large‐scale options like production‐grade bioreactors that can fulfil cGMP requirements will ultimately be the norm. Other manufacturing trends used routinely are automated operations, utilization of single‐use vessels and closed platforms, reducing the risk of contamination, and bringing online processing and process controls to the forefront. Large‐scale bioreactors are the technological advance that will allow industry to meet the challenges that the regulatory standards pose for hPSCs' use in clinical trials. The use of dynamic culturing systems for expansion of hPSCs assists researchers and companies bring their cell‐based therapies to the clinics faster, safer, and more efficiently.

## AUTHOR CONTRIBUTIONS

SET conceived, wrote, revised the manuscript, and made the figure and table. BER reviewed, revised, and edited the manuscript. Both authors read and approved the final manuscript.

## CONFLICT OF INTEREST

Benjamin Reubinoff is the CSO and holds shares in Cell Cure Neurosciences, Ltd.

## Data Availability

Data sharing is not applicable to this article as no new data were created or analyzed in this study.
